# Open-source QSAR models for pKa prediction using multiple machine learning approaches

**DOI:** 10.1186/s13321-019-0384-1

**Published:** 2019-09-18

**Authors:** Kamel Mansouri, Neal F. Cariello, Alexandru Korotcov, Valery Tkachenko, Chris M. Grulke, Catherine S. Sprankle, David Allen, Warren M. Casey, Nicole C. Kleinstreuer, Antony J. Williams

**Affiliations:** 1 Integrated Laboratory Systems, Inc., P.O. Box 13501, Research Triangle Park, NC 27709 USA; 2 Science Data Software LLC, 14914 Bradwill Court, Rockville, MD 20850 USA; 3 National Center for Computational Toxicology, U.S. Environmental Protection Agency, 109 T.W. Alexander Dr., Mail Code D143-02, Research Triangle Park, NC 27709 USA; 4 National Institute of Environmental Health Sciences, P.O. Box 12233, Mail Stop K2-16, Research Triangle Park, NC 27709 USA

**Keywords:** pKa prediction, QSAR, DataWarrior, Machine learning, Chemical 2D descriptors, Chemical fingerprints, PaDEL

## Abstract

**Background:**

The logarithmic acid dissociation constant pKa reflects the ionization of a chemical, which affects lipophilicity, solubility, protein binding, and ability to pass through the plasma membrane. Thus, pKa affects chemical absorption, distribution, metabolism, excretion, and toxicity properties. Multiple proprietary software packages exist for the prediction of pKa, but to the best of our knowledge no free and open-source programs exist for this purpose. Using a freely available data set and three machine learning approaches, we developed open-source models for pKa prediction.

**Methods:**

The experimental strongest acidic and strongest basic pKa values in water for 7912 chemicals were obtained from DataWarrior, a freely available software package. Chemical structures were curated and standardized for quantitative structure–activity relationship (QSAR) modeling using KNIME, and a subset comprising 79% of the initial set was used for modeling. To evaluate different approaches to modeling, several datasets were constructed based on different processing of chemical structures with acidic and/or basic pKas. Continuous molecular descriptors, binary fingerprints, and fragment counts were generated using PaDEL, and pKa prediction models were created using three machine learning methods, (1) support vector machines (SVM) combined with k-nearest neighbors (kNN), (2) extreme gradient boosting (XGB) and (3) deep neural networks (DNN).

**Results:**

The three methods delivered comparable performances on the training and test sets with a root-mean-squared error (RMSE) around 1.5 and a coefficient of determination (R^2^) around 0.80. Two commercial pKa predictors from ACD/Labs and ChemAxon were used to benchmark the three best models developed in this work, and performance of our models compared favorably to the commercial products.

**Conclusions:**

This work provides multiple QSAR models to predict the strongest acidic and strongest basic pKas of chemicals, built using publicly available data, and provided as free and open-source software on GitHub.

## Introduction

The acid dissociation constant (also called the protonation or ionization constant) Ka is an equilibrium constant defined as the ratio of the protonated and the deprotonated form of a compound. Ka is usually represented as pKa = − log10 Ka [1]. The pKa of a chemical strongly influences its pharmacokinetic and biochemical properties. pKa reflects the ionization state of a chemical, which in turn affects lipophilicity, solubility, protein binding, and ability to cross the plasma membrane and the blood–brain barrier.

The contributions of physicochemical parameters, including pKa, to environmental fate, transport, and distribution are well-recognized [2–5]. Chemicals with no charge at a physiological pH will cross the plasma membrane more easily than charged molecules and will therefore have greater potential for pharmacological or toxicological activity. Thus, pKa affects absorption, distribution, metabolism, excretion, and toxicity properties and is considered one of the five most important parameters in drug discovery [6, 7].

pKa is also an important parameter for physiologically based pharmacokinetic (PK) modeling and in vitro to in vivo extrapolation. Approaches such as those described by Wetmore et al. [8] are producing data sets that characterize metabolism and excretion for hundreds of chemicals. These data sets provide input for high-throughput methods for calculating the apparent volume of distribution at steady state and tissue-specific PK distribution coefficients [9] that will allow for the rapid construction of PK models. These, in turn, will provide context for both biomonitoring data and high-throughput toxicity screening studies.

Distribution of a chemical in an octanol/water mixture (described by the constants logKow or logP) is affected by the ionizable groups present in the chemical and is pH-dependent. logD is the distribution coefficient that takes into account the pH. This constant is therefore used to estimate the different relative concentrations of the ionized and non-ionized forms of a chemical at a given pH. Together, pKa and logP can be used to predict logD values [10]. This pH-dependent prediction is important to consider when attempting to predict absorption. For example, pH varies widely through the body from about 1.5 in the lower portion of the stomach to about 8.5 in the duodenum. Ionization characteristics of a chemical across this pH range therefore influence absorption in different locations in the body. The ability to predict logP and pKa and utilize these parameters to predict logD can therefore be of value for a number of applications, including drug design. The development of computational models to predict such physicochemical properties is clearly of value, quantitative structure–activity relationship (QSAR) models being one such approach.

Quantitative structure activity/property relationships (QSAR/QSPR) models for hydrophobicity were first developed in the 1960s [11]. The conceptual basis of QSARs is the congenericity principle, which is the assumption that structurally similar compounds will have similar properties. While QSAR approaches have been used for decades, their accuracy is highly dependent on data quality and quantity [12, 13]. Multiple commercial software vendors have developed systems for QSAR-based physicochemical parameter estimation, such as BioByte, ACD/Labs, Simulations Plus, ChemAxon and many others [14–17].

Different machine learning algorithms and variable selection techniques have been used in combination with molecular descriptors and binary fingerprints to develop QSAR models for physicochemical and toxicological properties. The advent of open data, open source, and open standards in the scientific community resulted in a plethora of web-based sites for sourcing data and performing real-time predictions. Examples include OCHEM, QSARDB, ChemBench and others [18–21].

As environmental scientists and modelers supporting U.S. government projects, our interest is in the development of free and open-source data and algorithms that are provided to the scientific community in such a way that more data can be incorporated, and additional models can be developed, consistent with government directives [22, 23]. Full transparency may also increase regulatory acceptance and confidence in modeling predictions.

pKa prediction is challenging because a single chemical can have multiple ionization sites. An examination of approximately 600 drugs showed that about 70% contain a single ionization site, with 45% of the compounds having a single basic ionization site and 24% having a single acidic site [24]. QSAR/QSPR methods generally perform better at predicting single endpoints. Consequently, many pKa models are restricted to small chemical spaces such as anilines, phenols, benzoic acids, primary amines, etc. [25, 26].

In addition, the paucity of large, freely available, high-quality, experimentally derived pKa datasets hinders the development of open-source and open data models. Indeed, both the quality of chemical structures and the associated experimental data are of concern due to their potential effects on the robustness of QSAR/QSPR models and the accuracy of their predictions [13, 27].

Several companies have developed algorithms to predict the pKa of individual ionization sites; several programs also predict multiple ionization sites for a single chemical [28]. However, to the best of our knowledge, there are no free, open-source, and open data models for predicting pKa for heterogeneous chemical classes. Liao and Nicklaus compared nine programs that predict pKa using a validation data set of 197 pharmaceuticals that included acetaminophen, aspirin, aspartame, ascorbic acid, amphetamine and many more well-studied chemicals [28]. However, it was highly likely that many of the chemicals used to assess model performance were also used to build some of the models, but lack of access to the underlying data precluded ascertaining this.

The aim of this work was to develop in silico models for the prediction of the most acidic and most basic pKa values for a chemical, rather than the values for all potential ionizable sites, and make both the data and models available as free and open-source tools.

The freely available pKa dataset used to develop our models was from the DataWarrior application [29]. The chemical structures were curated and standardized for modeling using a published, freely available workflow [13, 30]. Furthermore, the processing of duplicate chemical structures and amphoteric chemicals (chemicals that have both an acidic and basic pKa) was conducted in different ways (options 1, 2 and 3 explained here below) to provide several options for data modeling. The resulting QSAR-ready structures were used to generate 1D/2D chemical descriptors, binary fingerprints, and substructure counts using the freely available program PaDEL [31]. We then used three different modeling approaches—deep neural networks (DNN), support vector machines (SVM), and extreme gradient boosting (XGB)—to create the best possible models for pKa prediction.

All chemicals and associated experimental pKa values used to build and validate the models for this work are provided in Additional file 1. Open access to modeling data is extremely important for the scientific community to support continuous model improvement and to accurately assess model performance, in particular to avoid inflated statistics due to overlap of chemicals between training and validation sets.

## Materials and methods

### Data collection, curation, and preparation for modeling

#### The pKa data

The pKa data were obtained from the DataWarrior application [29, 32]. The DataWarrior file “pKaInWater.dwar” (available in the DataWarrior application folder) contains pKa data experimentally measured in water for 7912 chemicals. Chemical structures are provided as SMILES strings.

**Fig. 1:**
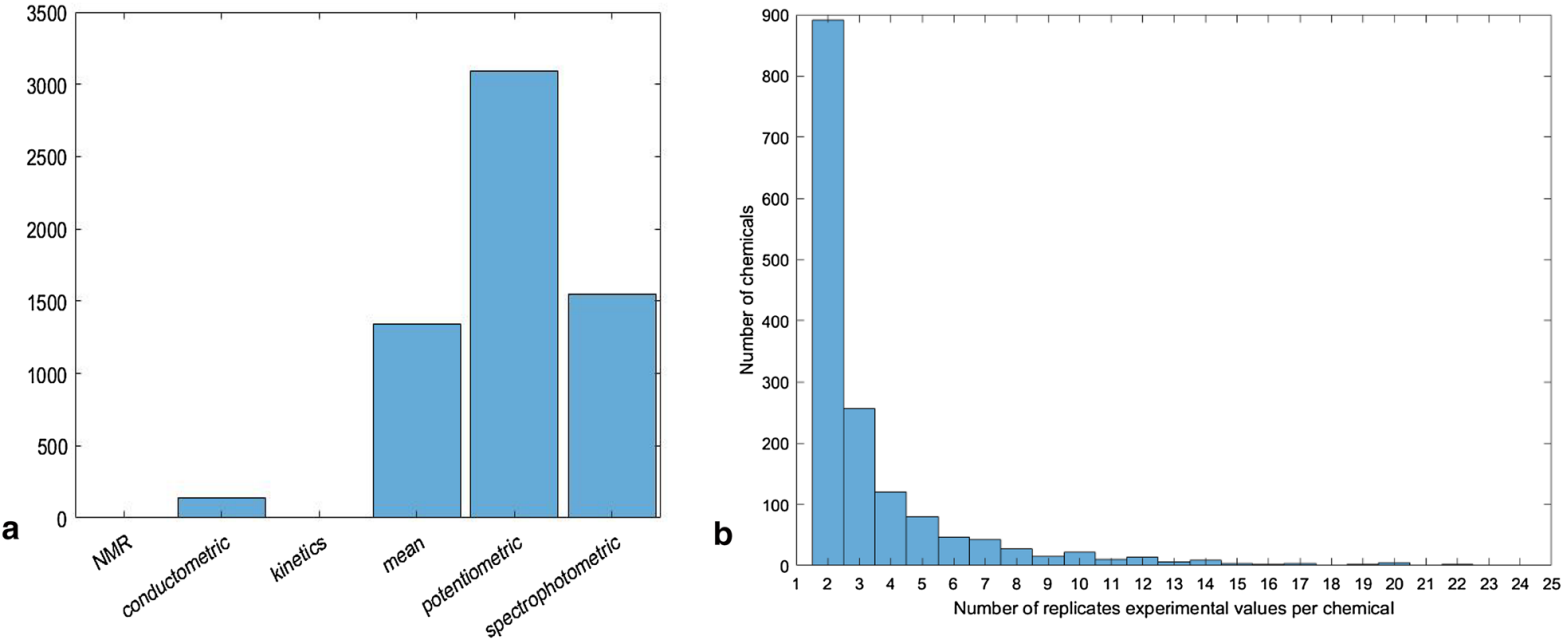
**a** Six methods of measuring pKa were used for the 7912 DataWarrior chemicals. Only four chemicals had pKas measured by NMR, and five chemicals had kinetic measurements of pKa, thus those bars are not visible in the histogram. No information on the experimental method used to determine pKa was provided for 901 chemicals. **b** Distribution of the number of chemicals having averaged experimental values

Of the 7912 chemicals in the data set, 1659 chemicals had both an acidic and basic pKa. Multiple acidic or multiple basic pKa protonation states for individual chemicals were not given. The collected chemical structures were analyzed for diversity using Toxprint chemotypes [33]. The enrichment graph (available in Additional file 2) shows the high diversity of the functional groups present in the dataset and is an indication of heterogeneity. The data were primarily collected from the literature by the DataWarrior providers but there are no references to support the pKa values. The file also contained information regarding methods used for the determination of the pKa values, as shown in Fig. 1a. Values of pKa provided for 1567 of the 7912 chemicals represent the mean of multiple experimental measurements (distribution in Fig. 1b).

To verify the accuracy of the data, chemicals having at least five pKa measurements were identified (Fig. 1) and 75 of these were randomly selected and compared to literature values. Literature pKa data were found for 80% of the chemicals and 93% of these chemicals were within ± 0.30 pKa units of the DataWarrior values. Considered this to indicate that the DataWarrior pKa values were sufficiently robust to support further investigation.

#### Curation of data and preparation for modeling

**Fig. 2:**
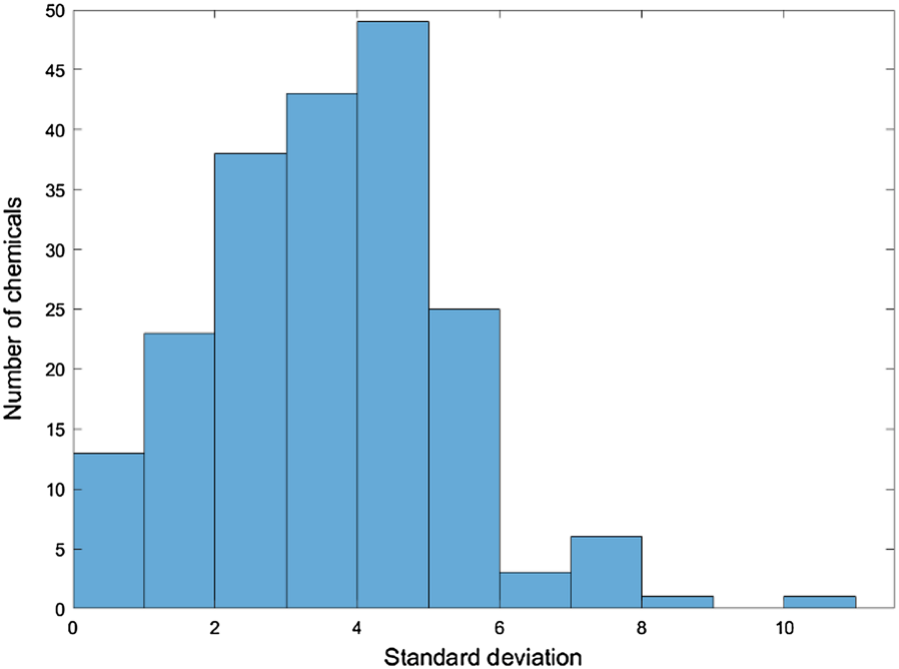
Distribution of standard deviations, in pKa units, for chemical structures with at least three replicate pKa values

The initial dataset of 7912 chemical structures had 3614 acidic pKa values and 4298 basic pKa values. A KNIME [34] workflow was used to standardize the structures and generate QSAR-ready forms for modeling [13, 27, 30, 35]. This workflow excludes inorganic chemicals and mixtures; removes salts, solvents, and counterions; identifies duplicates; and normalizes tautomers (e.g., nitro mesomers and keto-enol forms, zwitterions are not modified). This procedure yielded 6245 unique QSAR-ready structures. The deduplication of chemical structures was performed separately for the acidic and basic datasets. A total of 1659 chemical structures had two or more pKa values. Figure 2 shows the standard deviation distribution for the chemicals with at least three replicate values from both the acidic and basic data sets. This included the amphoteric chemicals (having both acidic and basic pKas) as well as additional duplicate structures where the pKa values were not averaged.

**Fig. 3:**
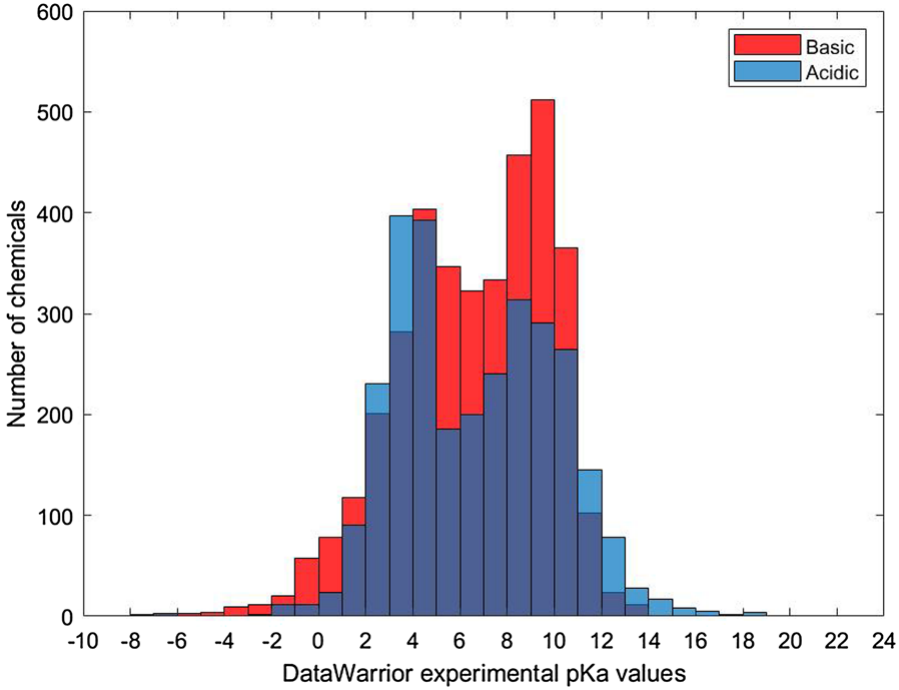
Distribution of the DataWarrior pKa values in the acidic and basic datasets

The final acidic data set consisted of 3260 unique QSAR-ready structures, and the basic data set had 3680 unique QSAR-ready structures. Figure 3 shows the distribution of pKa values for the acidic and basic data sets. This list was registered in the U.S. Environmental Protection Agency (EPA) DSSTox database using the EPA ChemReg chemical registration system to associate the chemical structures with valid identifiers such as CASRNs, DTXSIDs, and names (available in Additional file 3) [36]. In order to determine the optimal handling of chemicals with multiple differing pKa values, as well as the identification of amphoteric chemicals, three different data sets were constructed in different ways (described below as Options 1, 2 and 3). This provided different options of modeling the data for each approach.

##### Option 1: all chemicals with replicates removed

Chemicals with a single acidic or basic pKa and amphoteric chemicals with both one acidic and one basic pKa were selected. This yielded 6188 total chemicals, with 2960 having only a single acidic pKa, referred to as the acidic data set, and 3158 with only a single basic pKa, referred to as the basic data set. A third data set, called the combined data set, was generated by removing amphoteric chemicals. This data set consisted of 4897 chemicals with a single acidic or basic pKa. Option 1 was designed to avoid variability around pKa values.

##### Option 2: low variability replicates included

In order to include structures with low pKa variability, multiple values having an overall standard deviation of less than 2 pKa units were averaged. This increased the size of the acidic data set to 3095 structures, the basic data set to 3370 structures and the combined (non-amphoteric) data set to 5263 structures.

##### Option 3: all data included

For this last option, the entire QSAR-ready list of structures was used, including amphoteric chemicals. The acidic and basic data sets had 3260 and 3680 unique QSAR-ready structures, respectively. The pKa values of the replicates were averaged when the replicates collectively had a standard deviation of 1 pKa unit or less: otherwise, only the strongest acidic pKa (minimum value) and strongest basic pKa (maximum value) were included.

#### Training and test set preparation

Each of the three data sets described above was split into a training set (75%) and a test set (25%) in a semi-random way to keep a similar distribution of the pKa values. Thus, the training and test set were constructed to maintain a balance of the number of replicates in the two sets that were processed differently in each option as described above. The number of entries for the acidic, basic, and the amphoteric structures removed from the combined data sets (Option 1 and Option 2) was also similarly distributed between the training and test sets. This splitting approach avoided biasing the model toward a certain interval of the pKa values or towards one of the classes (acidic/basic) when modeling the combined data sets. Each of the different modeling approaches used the same training and test data sets corresponding to Options 1–3.

#### Chemical descriptors and fingerprints

The QSAR-ready structures were used to calculate molecular descriptors and generate binary fingerprints and fragment counts using the free and open-source tool PaDEL [31]. Because the original and standardized structures encoded 2D structural information, only 1D and 2D descriptors were calculated. The PaDEL output files contained 1444 continuous descriptors, 9121 binary fingerprints (CDK, Estate, CDK graph only, MACCS, PubChem, Substructure, Klekota-Roth and 2D atom pairs) and 5947 fragment counts (Substructure, Klekota-Roth and 2D atom pairs). Depending on the modeling approach, further filtering was employed to remove highly correlated features and near-zero variance features, and continuous descriptors were scaled.

### Machine learning algorithms

#### Support vector machines

SVM is a machine learning technique that was originally designed to solve classification problems but has since been generalized for application to continuous models such as those needed to predict pKa values. The SVM algorithm defines a decision boundary that optimally separates two classes by maximizing the distance between them [37, 38]. The decision boundary is a hyperplane that is a linear combination of functions parameterized by support vectors, which consist of a subset of training molecules.

Each of our three data sets was modeled separately using the free and open-source package LibSVM3.1 [39, 40]. Fivefold cross-validation was used to optimize model performance using the training data. Each model’s predictive ability was assessed using the external test sets. The fitting and cross-validation performance of the SVM models was evaluated using the coefficients of determination R^2^ and Q^2^, respectively [41, 42].

Since acidic and basic data sets were modeled separately, in order to predict pKa for a new chemical, it was necessary to decide whether the chemical had an acidic, basic, or amphoteric structure. A three-class categorical model was developed for this purpose. Genetic algorithms (GA) were used to find the optimal subset of molecular descriptors that differentiated the three categories of structures (acidic, basic and amphoteric). GA analysis began with an initial random population of chromosomes, which are binary vectors representing the presence or absence of molecular descriptors. Then an evolutionary process was simulated to optimize a defined fitness function, and new chromosomes were obtained by coupling the chromosomes of the initial population with genetic operations such as crossover and mutation [43, 44]. The fitness function used was the multiclass balanced accuracy (BA) calculated in a fivefold cross-validation procedure. Then the selected descriptors were applied to an SVM classifier as well as a k-nearest neighbors (kNN) approach (based on the majority vote of the nearest neighbors) in order to fit a classification model.

The best-performing continuous SVM models, which predicted pKa values, and the best-performing categorical SVM or kNN models, which predicted whether a chemical would have an acidic or basic pKa or be amphoteric, were selected and implemented in OPERA, a free and open-source suite of QSAR models [13, 27, 45]. OPERA’s global and local applicability domain (AD) approaches and its accuracy estimation procedure were applied to the acidic and basic pKa predictions [27]. The global AD is a Boolean index based on the leverage approach for the whole training set, while the local AD is a continuous index with a range from zero to one based on the most similar chemical structures from the training set [46]. Since binary fingerprints were employed to build the predictive models, the Jaccard–Tanimoto dissimilarity index was used as the distance metric to assess the AD and accuracy estimates.

The continuous molecular descriptors, as well as the binary fingerprints and fragment counts, were generated using version 2.21 of the free and open source tool PaDEL [31]. The LibSVM3.1 library used for this work was the C++ version developed by Chang et al. which is also available in other programming languages [39, 40]. The variable selection using the GA to build the SVM models, calling the C++ LibSVM code, and kNN models were performed in MATLAB 2018a [47].

The final kNN/SVM models were implemented in the free and open source OPERA application (version 2.0) that is available on Github at: https://github.com/NIEHS/OPERA.

#### Extreme gradient boosting

Gradient boosting is a machine learning technique for regression and classification problems. It produces a prediction model that represents a compilation of weak prediction models, typically decision trees. Gradient boosting builds the weak models in a stage-wise fashion and generalizes them by allowing optimization of an arbitrary differentiable loss function.

XGB is an extension of gradient boosting that prevents overfitting by using an improved cost function [48–50]. A QSAR XGB model showed very good performance when analyzing 30 pharmaceutical datasets, including inhibition of CYP450, hERG channel, and several ion channels [51].

We used the R package caret with the R implementation of XGB and the xgbLinear option. Importantly, the caret implementation performs model tuning and calculates variable importance [52, 53]. R version 3.5.0 for Windows, xgboost version 0.6.4.1, and caret package version 6.0.79 were used for the XGB modeling. While many other machine learning algorithms could have been used, XGB was deemed to be a reasonable place to start for comparison of the PaDEL binary fingerprints, fragment count, and 1D/2D descriptors.

Root-mean-squared error (RMSE) was optimized using the training data with fivefold cross validation repeated five times. The acidic and basic data sets were modeled separately. Each of the three data sets (Options 1–3) was examined and performance was assessed for the testing data sets using RMSE and the coefficient of determination R^2^. In addition, three feature-reduction techniques were examined to assess impact on model performance of using: (1) data in which features (columns) of all zeros and all ones were deleted, (2) as previous but with highly correlated features removed, and (3) as previous but with low-variance features removed.

An RData environment file was generated for all the XGB models. The RData file can be loaded into the R workspace to quickly access all models and variables. The RData environment and performance metrics are found on [54]. R Markdown was used to create a HTML file with all the performance metrics, variable importance plots and R^2^ plots. Additional XGB details are in Additional file 2 and in the code documentation on the GitHub site.

#### Deep neural networks

DNN learning has been used extensively in computational biology [55–57] and computational chemistry [58–60]. A DNN learning model consists of artificial neural networks with multiple layers between the input and the output. One significant advantage of using DNN learning is that it maximizes the model accuracy by mapping features through a series of nonlinear functions that are stitched together in a combinatorial fashion.

The DNN learning models were built using the open-source deep learning libraries Keras 2.0 and Tensorflow 1.4 [61, 62]. The open source Scikit-learn Python library was used for feature vector processing, fivefold cross validation, and final metric computations [63]. Python 3.6 was used for all DNN coding using a Jupyter notebook.

Fivefold cross validation was used to construct a model from the training data by optimizing RMSE. A variety of parameters were examined and optimized, including the algorithm, weight initialization, hidden layers activation function, L2 regularization, dropout regularization, number of hidden layers, nodes in the hidden layers, and the learning rate.

**Fig. 4:**
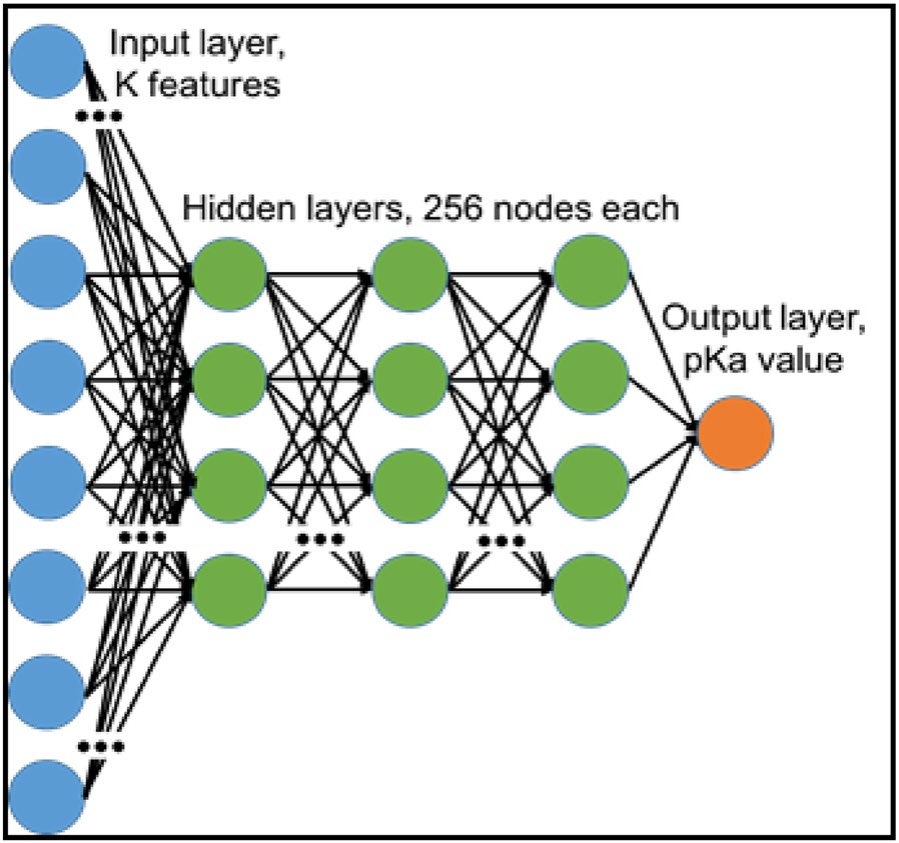
DNN learning model for pKa prediction. The model was comprised of a four-layer neural network with one input layer (K features), three hidden layers (256 nodes each) and one output layer (pKa value). Each hidden layer was followed by a batch normalization layer and a dropout layer (not shown). Connections existed between neurons across layers, but not within a layer

DNN models were trained using all binary, count fingerprints, 1D/2D descriptors, and their combinations. 1D/2D features that had any missing values were excluded. All feature vectors with continuous variables were scaled to absolute values of minimum and maximum values prior to training. The final tuned model had three hidden layers of 256 nodes each followed by a batch normalization and a dropout layer (0.25). The overall architecture is shown in Fig. 4.

#### Benchmarking the developed models

To further validate the three models and assess their predictivity, a large external data set that was not used during the modeling process would be ideal. However, no large, well-annotated pKa datasets were found in the literature. Thus, in lieu of experimental data, the possibility of benchmarking the models using predictions that could be verified to be consistent with DataWarrior was tested.

We selected two widely used commercial predictors from ACD/Labs and ChemAxon to be used in the benchmark analysis. Both ACD/Labs and ChemAxon have multiple pKa predictors and models. For ACD/Labs Percepta Batch (version 2016.2.2), the “Single_Classic” model was selected with Amides and S-Acids considered as non-ionizable. For ChemAxon, the strongest acidic and basic pKa values were considered.

First, ACD/Labs and ChemAxon pKa predictions were compared to the DataWarrior pKa values. This tested the hypothesis that predictions generated by the two commercial tools were concordant enough (either separately or in combination) with the experimental DataWarrior data set to be used as benchmarks for the three models. The concordance metrics were the number of chemicals commonly predicted to have acidic and basic pKas as well as the statistical parameters: R^2^, coefficient of correlation (r^2^), and RMSE. For this first step of the analysis, ACD/Labs and ChemAxon predictions were generated using the same QSAR-ready standardized structures generated from the DataWarrior chemicals and used to develop the models. This concordance analysis used data Option 3, which includes amphoteric chemicals, mean pKa values for replicates, and the strongest acidic pKa (smallest value) or strongest basic pKa (greatest value).

This concordance analysis had two main goals. The first was to identify a set of rules or chemical space/pKa ranges where these two tools were most concordant with DataWarrior pKa values. These rules would then be applied to predictions from ACD/Labs and ChemAxon on a new data set to generate benchmark data to compare with the predictions of the three models developed in this work. A subset of the EPA Toxic Substances Control Act (TSCA) chemicals was used for this purpose. All predictions in this analysis were based on QSAR-ready structures generated using the previously mentioned structure standardization procedure.

## Results and discussion

### Support vector machines

The above described datasets from Options 1–3 were modeled using the SVM algorithm, and the results are shown in Table 1. The acidic and basic datasets were modeled separately using continuous descriptors, binary fingerprints, fragment counts, and combined binary fingerprints-fragment counts.

**Table 1:** Performance of SVM models using three data options with continuous descriptors, fingerprints and fragment counts

Data option	Data set	Feature sets	Number of features	Train		Fivefold CV		Test	
				R^2^	RMSE	Q^2^	RMSE	R^2^	RMSE
1	Acidic	Continuous	870	0.96	0.65	0.58	2.18	0.68	1.91
1	Acidic	Fingerprints	1548	0.91	1.00	0.64	2.02	0.71	1.81
1	Acidic	Fingerprints + counts	2104	0.94	0.80	0.64	2.02	0.72	1.80
1	Basic	Continuous	876	0.96	0.64	0.65	1.94	0.65	1.93
1	Basic	Fingerprints	1535	0.91	0.99	0.69	1.84	0.69	1.83
1	Basic	Fingerprints + counts	2079	0.93	0.87	0.72	1.73	0.70	1.80
2	Acidic	Continuous	913	0.98	0.49	0.61	2.10	0.69	1.89
2	Acidic	Fingerprints	1552	0.9	1.05	0.63	2.04	0.69	1.87
2	Acidic	Fingerprints + counts	2141	0.94	0.85	0.63	2.05	0.71	1.81
2	Basic	Continuous	913	0.97	0.52	0.67	1.88	0.66	1.88
2	Basic	Fingerprints	1534	0.90	1.02	0.68	1.83	0.75	1.63
2	Basic	Fingerprints + counts	2085	0.93	0.88	0.71	1.76	0.78	1.53
3	Acidic	Continuous	510	0.96	0.66	0.59	2.17	0.57	2.20
3	Acidic	Fingerprint	1580	0.91	1.00	0.64	2.01	0.68	1.91
3	Acidic	Fingerprints + counts	2395	0.93	0.86	0.65	1.99	0.69	1.87
3	Basic	Continuous	510	0.95	0.75	0.61	2.01	0.6	2.09
3	Basic	Fingerprints	1543	0.91	0.94	0.72	1.72	0.67	1.90
3	Basic	Fingerprints + counts	2358	0.93	0.84	0.73	1.67	0.71	1.79

The acidic dataset from Option 1 with fingerprints and fragment counts showed the best performance on the test set, with an R^2^ of 0.72 and an RMSE of 1.80. Among the SVM models predicting basic pKa, the dataset from Option 2 with fingerprints and fragment counts showed the best overall performance, with a test set R^2^ and RMSE of 0.78 and 1.53, respectively. The continuous 1D/2D descriptors performed poorly, while the models using binary fingerprints combined with fragment counts showed the best overall performance. In general, the basic pKa models performed better than the acidic pKa models for the three data options.

Since the pKa value prediction should be combined with a decision algorithm to decide whether to use the acid or basic model or both, the classification modeling described above was used. First the GA identified 15 continuous descriptors of relevance in differentiating acidic, basic, and amphoteric chemicals (Table 2). Each of these descriptors is related to the electronic configuration of the structures and their H-bond donors/acceptors and thus can be interpreted as mechanistically linked to pKa. Then, these descriptors were used to calibrate a three-class kNN categorical model. In order to challenge the kNN model based on the 15 GA selected continuous descriptors, its performance was compared to SVM models based on the same descriptors as well as fingerprints and fragment counts.

**Table 2:** Descriptors selected by the genetic algorithm to differentiate chemicals with acidic and/or basic ionization sites

Name	Description
minHBa	Minimum e-states for (strong) hydrogen bond acceptors
minsOH	Minimum atom-type e-state: –OH
TopoPSA	Topological polar surface area (TPSA)
WTPT4	Sum of path lengths starting from oxygens
WTPT5	Sum of path lengths starting from nitrogens
SaaN	Sum of atom-type e-state: N
SsOH	Sum of atom-type e-state: –OH
minHBint2	Minimum e-state descriptors of strength for potential hydrogen bonds of path length 2
AATS0i	Average Broto-Moreau autocorrelation − lag 0/weighted by first ionization potential
ATSC1i	Centered Broto-Moreau autocorrelation − lag 1/weighted by first ionization potential
maxHBa	Maximum e-states for (strong) hydrogen bond acceptors
nHBAcc	Number of hydrogen bond acceptors (using CDK HBondAcceptorCountDescriptor algorithm)
nHBint2	Count of e-state descriptors of strength for potential hydrogen bonds of path length 2
ETA_dBeta	A measure of relative unsaturation content
nHBDon_Lipinski	Number of hydrogen bond donors (using Lipinski’s definition: any OH or NH, each available hydrogen atom is counted as one hydrogen bond donor)

The results, summarized in Table 3, confirmed that the kNN model based on the best 15 descriptors is more robust and stable in comparison to the other models.

**Table 3:** Comparison of kNN classification model and SVM models

Model algorithm	Descriptor type	Variables	Train BA	Fivefold CV BA	Test BA
kNN	Continuous	15	0.8	0.8	0.77
SVM	Continuous	15	0.92	0.8	0.73
SVM	Continuous	511	0.98	0.79	0.72
SVM	Fingerprints	1565	0.98	0.8	0.74
SVM	Fragment counts	815	0.96	0.8	0.73

The kNN classification model used the 15 GA selected descriptors. The SVM models used the same descriptors as well as additional continuous descriptors, fingerprints, and fragment counts as noted in the table

Based on these results, a free and open-source pKa predictor was implemented in OPERA (since version 2.0) to be used with new chemicals [27, 35, 45]. The kNN classification model based on the 15 descriptors selected by GA is used to select the appropriate SVM model, which is then applied to predict the pKa values. The OPERA pKa predictor is also equipped with an ionization checker based on the hydrogen donor and acceptor sites such that pKa predictions will only be made for ionizable chemicals.

### Extreme gradient boosting

Three feature-reduction techniques were applied to the binary fingerprints and fragment counts descriptors:

- Data in which constant features (of all zeros and all ones) were deleted: D1.
- As above, but with highly correlated features removed: D2.
- As above, but with low variance features removed: D3.

Model performance and variable importance for all feature sets is available in Additional file 2. The performance for the five best models for the acidic and basic data sets is summarized in Table 4. The models for the best acidic and basic data sets had equivalent performance, with RMSEs of 1.68 and 1.69, respectively.

**Table 4:** Summary statistics for the five best-performing XGB models for chemicals with acidic and basic pKas

Data option	Dataset	Feature sets	Number of features	Train		Test	
				R^2^	RMSE	R^2^	RMSE
1	Acidic	Fingerprints (D1)	4901	0.684	1.865	0.754	1.679
1	Acidic	Fingerprints (D2)	4234	0.673	1.897	0.739	1.728
1	Acidic	MACCS (D2)	145	0.658	1.951	0.725	1.775
1	Acidic	Fingerprints (D3)	1663	0.655	1.948	0.710	1.825
1	Acidic	MACCS (D1)	153	0.657	1.953	0.706	1.834
2	Basic	Fingerprints (D2)	4009	0.752	1.540	0.728	1.694
2	Basic	Fingerprints (D1)	4665	0.749	1.551	0.723	1.709
2	Basic	PUBCHEM (D2)	488	0.727	1.622	0.720	1.718
2	Basic	MACCS (D3)	98	0.714	1.663	0.714	1.736
2	Basic	MACCS (D1)	153	0.734	1.601	0.712	1.744

Each group of statistics is ordered by test set RMSE, with the best-performing models listed first. D1 indicates the data set with variables of all 0’s and all 1’s removed. D2 indicates the D1 data set with highly correlated variables removed. D3 indicates the D2 data set with low variance features removed

In addition to modeling all eight binary fingerprints separately, another data set was created that combined the eight binary fingerprints. The best performance was obtained with the combined fingerprints. This is not surprising because the combined fingerprint data set allows the most informative features of any binary fingerprint to be used in the model. This approach performed better than use of any single binary fingerprint, fragment count, or 1D/2D descriptor. The MACCS fingerprint was the best performing single fingerprint.

R was used for the XGB analysis and R Markdown was used to create a single HTML file with all the performance metrics for all binary fingerprints, all counts, and 1D/2D data. Variable importance plots and observed vs. predicted R^2^ plots were generated for all models. The R workspace environment was saved for all models so the code does not have to be executed to examine the models. The user can simply load the R workspace into the current session.

### Deep neural networks

The three data set options (Option 1 and Option 2) were modeled separately using DNN. The results in Table 5 show that the model for chemicals with a single acidic pKa had the best performance, followed by chemicals with a single basic pKa and finally by chemicals with a single acidic and single basic pKa combined. Performance was measured using the RMSE for the test data. Models using data Options 1 and 2 outperformed models using data Option 3.

**Table 5:** Summary statistics for the five best-performing DNN models

Data option	Dataset	Feature sets	Number of features	Train		Fivefold CV		Test	
				R^2^	RMSE	Q^2^	RMSE	R^2^	RMSE
1	Acidic	Continuous + MACCS	1408	0.98	0.43	0.75	1.71	0.80	1.51
2	Acidic	Continuous + MACCS	1408	0.98	0.52	0.74	1.73	0.79	1.54
2	Acidic	Fingerprints	1190	0.98	0.48	0.71	1.82	0.79	1.55
1	Acidic	Fingerprints	1190	0.99	0.39	0.71	1.81	0.78	1.59
2	Acidic	MACCS	166	0.96	0.64	0.71	1.82	0.77	1.61
2	Basic	Fingerprints	1190	0.98	0.48	0.75	1.63	0.77	1.57
1	Basic	MACCS	166	0.97	0.53	0.74	1.69	0.77	1.59
1	Basic	Continuous + MACCS	1481	0.98	0.45	0.75	1.64	0.76	1.59
2	Basic	Continuous + MACCS	1481	0.97	0.56	0.73	1.71	0.76	1.60
2	Basic	MACCS	166	0.97	0.58	0.75	1.65	0.74	1.65
1	Combined	Continuous + MACCS	1408	0.97	0.52	0.65	1.90	0.75	1.61
1	Combined	Fingerprints	1190	0.97	0.55	0.62	1.98	0.73	1.68
2	Combined	Continuous + MACCS	1408	0.97	0.55	0.67	1.84	0.72	1.69
1	Combined	MACCS	166	0.97	0.57	0.62	1.99	0.72	1.70
2	Combined	MACCS	166	0.97	0.52	0.63	1.94	0.70	1.76

Statistics are presented for the acidic only, basic only and combined (acidic and basic) data sets. Each group of statistics is ordered by test set RMSE, with the best-performing models listed first

In all cases, models constructed using a combination of features outperformed models using a single fingerprint set. For the chemicals with an acidic pKa, the best-performing model combined 1D/2D descriptors and MACCs fingerprints using the Option 1 data. For the chemicals with a basic pKa, the best-performing model combined the MACCs and CDK fingerprints using the Option 2 data. For the data set that combined the chemicals with an acidic and basic dataset, the best performance was seen using the 1D/2D descriptors with the MACCS fingerprint.

### Comparison of SVM, DNN, and XGB model performance

Table 6 shows the RMSE and R^2^ statistics for the DNN, SVM, and XGB models with the best performance. Based on RMSE, the DNN model for chemicals with an acidic pKa was substantially better than the SVM and XGB models. However, the SVM model was marginally better than the DNN model for chemicals with a basic pKa.

**Table 6:** Summary statistics for the best-performing models using the testing data

Model	Acidic				Basic			
	Data option	Feature set	R^2^	RMSE	Data option	Feature set	R^2^	RMSE
SVM	1	Fingerprints + counts	0.72	1.80	2	Fingerprints + counts	0.78	1.53
XGB	1	Fingerprints	0.75	1.68	2	Fingerprints	0.73	1.69
DNN	1	Continuous + MACCS	0.80	1.51	2	Fingerprints	0.77	1.57

It is not clear why the DNN model for chemicals with an acidic pKa performed so well, as DNNs are notoriously difficult to interpret [64]. While DNNs have shown remarkable performance in many areas, in many cases they remain a black box [65]. For example, in our relatively small data set, there were 438,273 trainable parameters, which illustrates the complexity of many DNN models.

One important difference among the models is that the SVM models are coupled with a categorical model that can indicate whether a molecule has an acidic pKa, basic pKa or both (amphoteric). This leads to an automatic selection of the model to use (acidic, basic or both), for ionizable chemicals only, by OPERA models.

### Benchmark analysis

#### Concordance of pKa predictions from ACD/Labs and ChemAxon to the DataWarrior values

The QSAR-ready standardized structures generated from the DataWarrior chemicals were used to generate pKa predictions using the proprietary ACD/Labs Percepta Batch (version 2016.2.2) and ChemAxon predictors. The entire DataWarrior list (Option 3) was used as input for the two commercial tools to predict whether a chemical would have an acidic or basic pKa as well as to predict numeric pKa values. These tools can also provide multiple acidic and basic pKa values for a single chemical. However, for this study, only the strongest acidic and the strongest basic “macroscopic” pKas were considered. The predictions of both tools are provided in Additional file 4.

This comparison was conducted to analyze the concordance between DataWarrior and the predictions of ACD/Labs and ChemAxon. Thus, the goal was not to assess the predictive performance of the commercial tools.

Table 7 summarizes the total number of chemicals that were predicted to have acidic or basic pKas by the two commercial tools using the 6940 DataWarrior chemicals (Option 3). As shown in Table 7, the commercial tools provided pKa values for the overwhelming majority of the DataWarrior chemicals. Only 3.5% and 0.3% of the chemicals were predicted to be non-ionizable by ACD/Labs and ChemAxon, respectively. These numbers are substantially higher than the number of acidic and basic pKa values available from DataWarrior. The number of chemicals predicted as amphoteric by the commercial tools is also higher than what is available in DataWarrior’s experimental data.

**Table 7:** Acidic and basic pKas predicted by ACD/Labs and ChemAxon models using the DataWarrior chemicals

	Chemicals predicted to have an acidic pKa	Chemicals predicted to have a basic pKa	Chemicals predicted to be amphoteric	Chemicals predicted to be non-ionizable
ACD/Labs	4475	5210	2989	244
ChemAxon	5070	6447	4595	18
In common	4468	5031	2818	6

ACD/Labs seemed to be more selective than ChemAxon in terms of acidic/basic classification, while ChemAxon considered most of the chemicals it predicted as ionizable to be amphoteric. The summary data presented in Table 7 suggests that the two commercial tools are employing different algorithms to determine ionization sites and to classify the pKa of the chemicals as acidic and basic. However, the two tools also show a high number of chemicals predicted in both the acidic and basic categories (third row of Table 7).

**Fig. 5:**
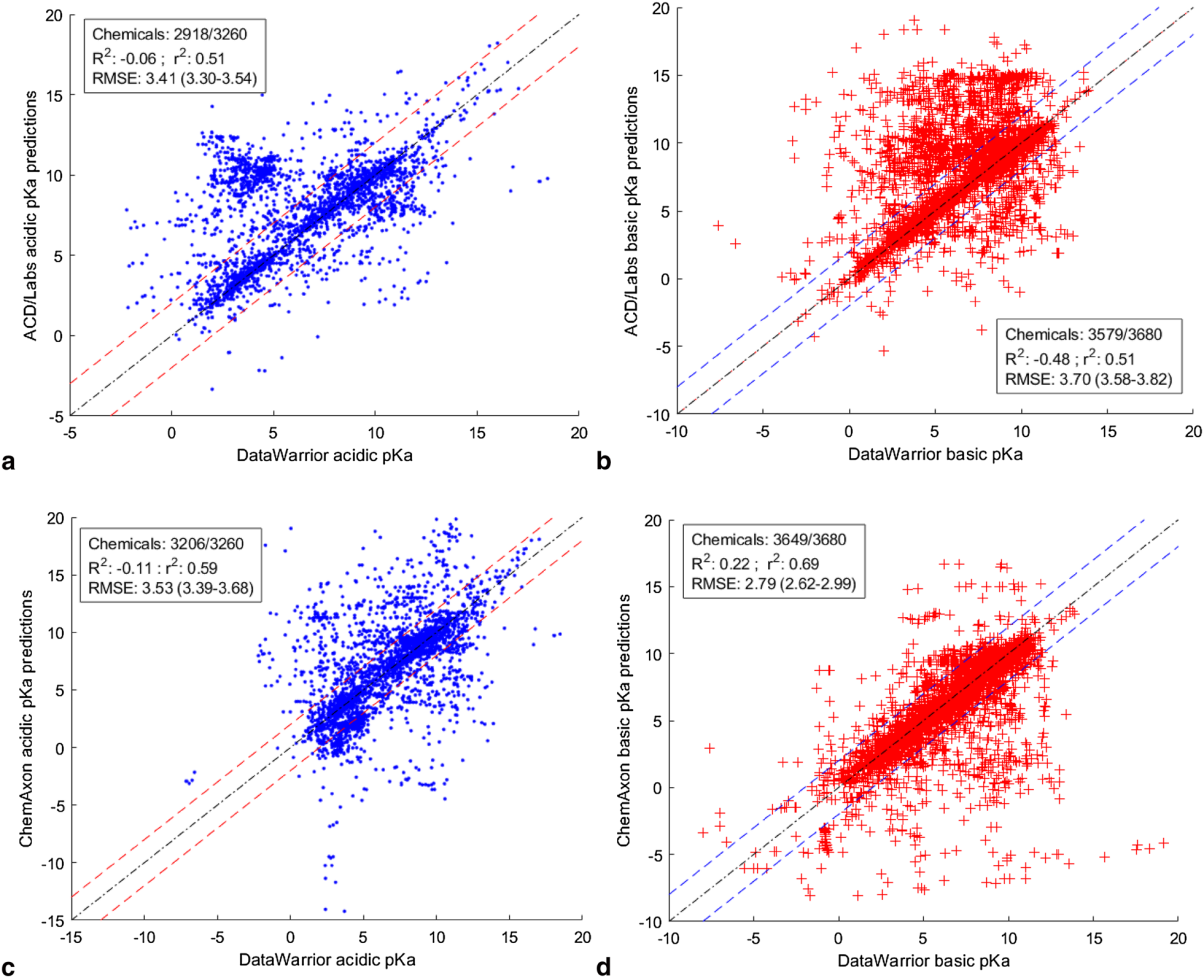
Scatter plots of ChemAxon and ACD/Labs predictions versus the DataWarrior acidic and basic pKa values. Dotted lines show the predictions for ChemAxon and ACD/Labs that are within± 2 pKa units of the DataWarrior values. **a** DataWarrior acidic pKa values vs ACD/Labs acidic pKa prediction. **b** DataWarrior basic pKa values vs ACD/Labs basic pKa prediction. **c** DataWarrior acidic pKa values vs ChemAxon acidic pKa prediction. **d** DataWarrior basic pKa values vs ChemAxon acidic pKa prediction. Values between parenthesis are the 95% confidence intervals based on a 5000-bootstrapping procedure

Next, the ACD/Labs and ChemAxon predictions were compared to the experimental pKa values available in DataWarrior. For each of the commercial tools, the concordance analysis was conducted on the chemicals in common with DataWarrior’s acidic and basic data sets separately. The results of this analysis are shown in Table 8 and Fig. 5. Over 90% of the DataWarrior chemicals with an acidic pKa were predicted to have an acidic pKa by both ACD/Labs and ChemAxon. Likewise, over 97% of the DataWarrior chemicals with a basic pKa were predicted to have a basic pKa by both ACD/Labs and ChemAxon. Thus, there is a high degree of overlap between both ACD/Labs and ChemAxon tools with DataWarrior acidic and basic sets in terms of number of predicted chemicals. However, as mentioned above, it is important to note that the two commercial tools predict a higher number of amphoteric chemicals than was indicated by the DataWarrior experimental data.

**Table 8:** Summary of the overlap between ChemAxon and ACD/Labs predictions

	Acidic dataset in DataWarrior (3260 chemicals)			Basic dataset in DataWarrior (3680 chemicals)		
	ACD/Labs	ChemAxon	In common	ACD/Labs	ChemAxon	In common
Number of predicted chemicals	2918	3206	2917	3579	3649	3557
pKa difference ≤ 2 units	1864	2022	1534	2178	2928	1974
pKa difference > 2 units	1054	1184	655	1401	721	445

Statistics are relative to the total number of predicted DataWarrior chemicals (QSAR-ready standardized SMILES with the Option 3 data)

Figure 5 plots the pKa predictions of the two commercial tools in comparison to the DataWarrior acidic and basic pKa data sets for the chemicals in common (Table 8). The concordance statistics of the predictions of those chemicals are also provided in the figure inserts as R^2^, r^2^, and RMSE. The data show moderate r^2^ correlations (0.51–0.69) but a low predictivity demonstrated by low R^2^ and high RMSE. However, Fig. 5 also shows that these low statistics are not representative of all plotted predictions. In fact, the dotted lines in the Fig. 5 graphs show that the number of predictions for both ACD/Labs and ChemAxon within a ± 2 pKa unit threshold is considerably greater than those above 2 pKa units difference with DataWarrior. This is confirmed in Table 8, which also shows that the two commercial tools show high concordance with DataWarrior in terms of the number of predictions within 2 pKa units error.

**Fig. 6:**
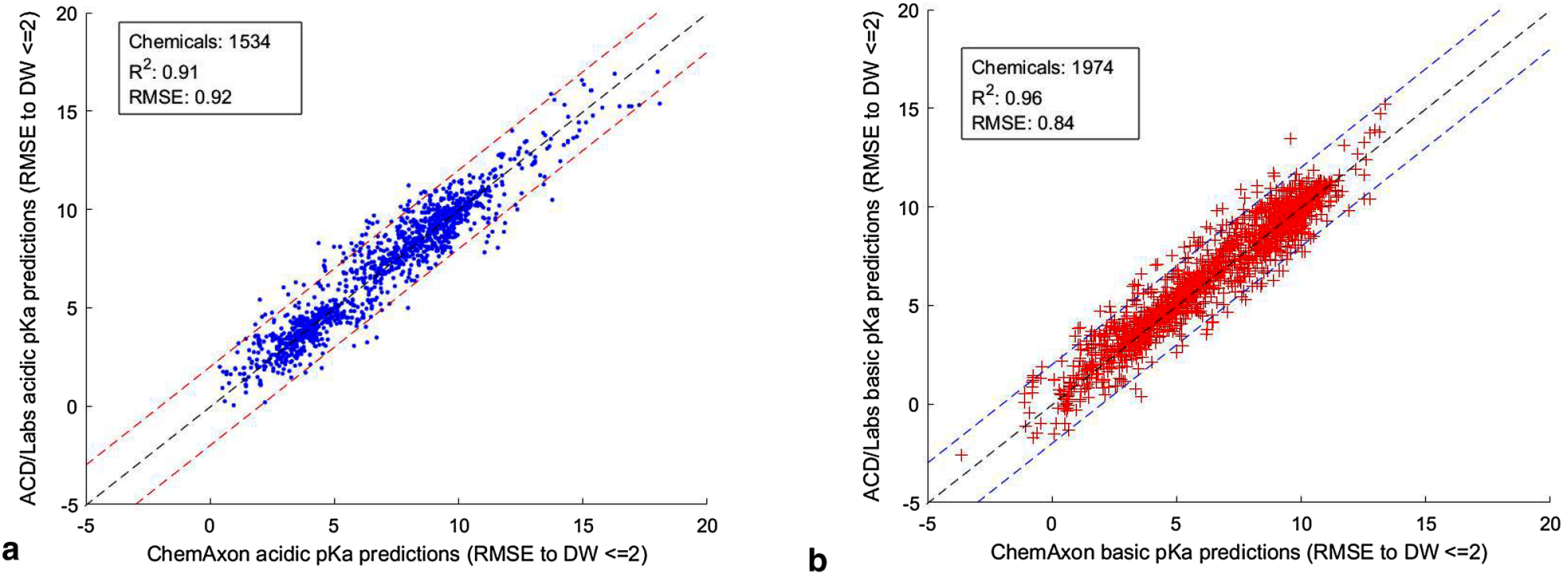
Scatter plots of ChemAxon and ACD/Labs predictions for the chemicals in common within 2 pKa units of the acidic and basic data sets of DataWarrior. **a** ChemAxon acidic pKa predictions vs ACD/Labs acidic predictions. **b** ChemAxon basic pKa predictions vs ACD/Labs basic predictions

Table 8 also shows a high overlap between the two tools in terms of the number of chemicals that are predicted to be within ± 2 pKa units of the DataWarrior values. This means, that for the most part, the two predictors are reasonably concordant (based on the 2 pKa units cutoff) with each other as well as with DataWarrior, as shown in Fig. 6. A structural comparison of the commonly predicted chemicals with an error of ≤ 2 and > 2 pKa units of the DataWarrior values did not reveal any trends in chemical features in the two groups. Thus, it seems that the differences between the two programs is multifaceted, with potential sources of variation for both commercial tools and DataWarrior including the prediction algorithms, data sources, and curation processes.

Considering only the ACD/Labs and ChemAxon predictions within 2 pKa units of the DataWarrior values, Fig. 6 shows that the commercial products have very high concordance, with an R^2^ > 0.91 and RMSE values below 0.92 for these chemicals. Thus, a ± 2 pKa units difference between ACD/Labs and ChemAxon predictions can be considered a reasonable threshold to include most of their predictions that are also concordant with DataWarrior. To verify this hypothesis, the number of chemicals that both tools predicted within ± 2 pKa units of each other and DataWarrior pKa values are summarized in Table 9. The high overlap between the two groups of chemicals for both acidic and basic data sets confirms that if both tools predicted a pKa value for a chemical within ± 2 pKa units of each other, those predictions are most likely to also be within ± 2 pKa units of the DataWarrior experimental pKa value. This is valid for both acidic and basic pKas.

**Table 9:** Concordance of ACD/Labs and ChemAxon pKa predictions with each other and DataWarrior values

	ACD/Labs and ChemAxon predictions within 2 pKa units of each other	Predictions within 2 pKa units of the DataWarrior pKa values	Overlap
ACD/Labs and ChemAxon acidic pKa predictions	1866	1543	1468
ACD/Labs and ChemAxon basic pKa predictions	2199	1974	1902

Table summarizes pKa predictions for ACD/Labs and ChemAxon chemicals within ± 2 pKa units of each other, within ± 2 pKa units to DataWarrior pKa values and the overlap between the two groups

**Fig. 7:**
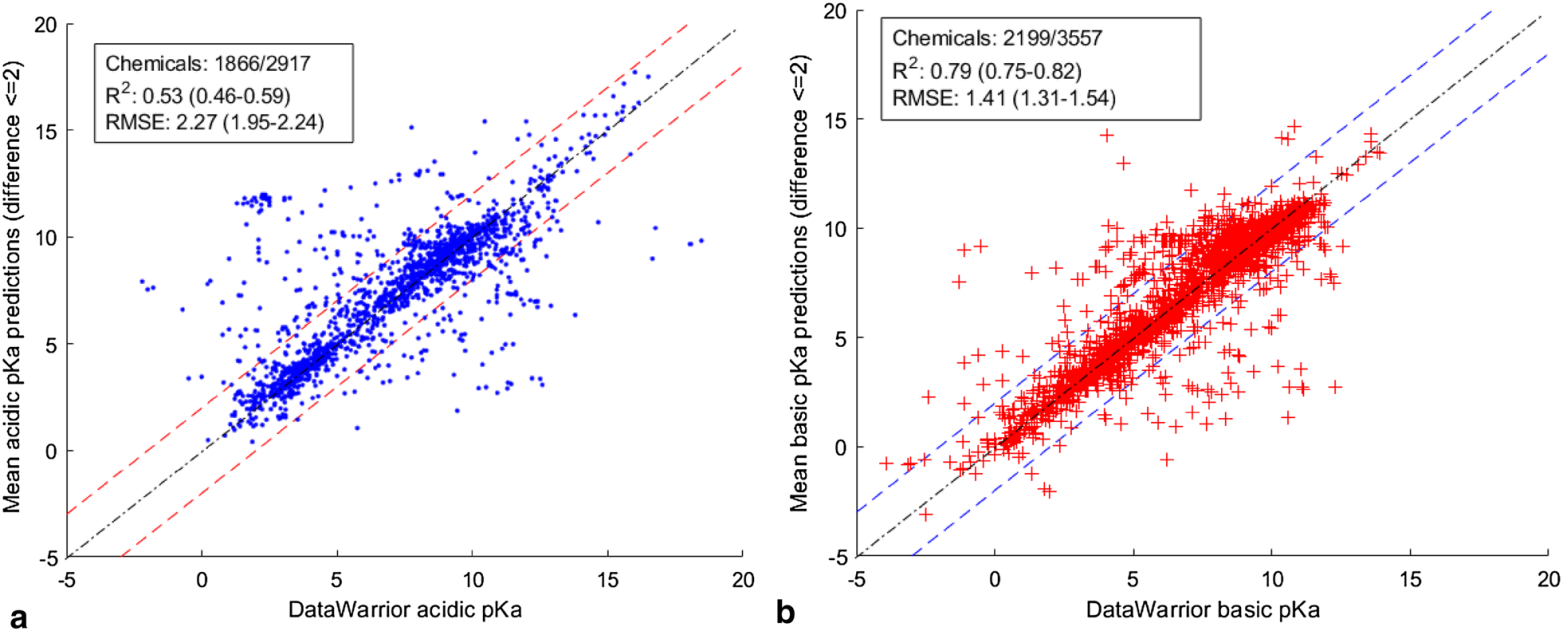
Scatter plots of the averaged ChemAxon and ACD/Labs predictions within ± 2 units of each other for the chemicals in the **a** acidic and **b** basic pKa data sets of DataWarrior values between parenthesis are the 95% confidence intervals based on a 5000-bootstrapping procedure

These results indicate that when the predicted pKa values using ACD/Labs and ChemAxon are within ± 2 pKa units of each other, these values are within the same threshold of difference with the DataWarrior experimentally measured pKa values. Conversely, when the predicted pKa values using ACD/Labs and ChemAxon are > 2 pKa units of each other, the concordance with the DataWarrior values is low. This suggests that the concordant pKa predictions (within ± 2 units of each other) can be averaged and used as a benchmark for our three models on a new data set. Figure 7 shows good concordance between the averaged predictions and the acidic and basic pKa values of DataWarrior.

#### Performance of the three models in comparison to the benchmark data

A subset of chemicals from the EPA Toxic Substances Control Act (TSCA) called “TSCA_active” (referred to simply as TSCA chemicals in this publication) were selected as a benchmark for the analysis. This dataset was downloaded from EPA’s CompTox Chemicals Dashboard [66] and yielded 9835 QSAR-ready structures after processing using the same standardized workflow applied to the DataWarrior chemicals. The DataWarrior data set included 931 of these chemicals, so these were removed, leaving 8904 chemicals for further analysis. The TSCA dataset did not have any experimental pKa values. The same ACD/Labs and ChemAxon models used previously were applied to this list of chemicals. The total number of predicted chemicals by the two commercial tools and the overlap between them are summarized in Table 10. All predictions for this dataset are provided in Additional file 5.

**Table 10:** Comparison of pKa predictions for the TSCA chemicals

	Acidic pKa predictions	Basic pKa predictions	Non-ionizable
ACD/Labs	3256	2490	4030
ChemAxon	4271	7208	1059
Chemicals in both ACD/Labs and ChemAxon predictions	3234	2366	1023
Difference ≤ 2 pKa units	2457	1089	–

Table 10 shows that there is considerable divergence between ACD/Labs and ChemAxon predictions with regard to the number of ionizable and non-ionizable chemicals. ACD predicted that 45% (4030/8904) of the chemicals would be non-ionizable, while ChemAxon predicted that 12% (1059/8094) of the TSCA chemicals would be non-ionizable. Note that for the ACD/Labs analysis, the option to consider amides and s-acids (~ 500 chemicals) as non-ionizable was selected. This divergence was greatest for the chemicals with a basic pKa. This was also reflected in the concordance between the two tools in terms of predicted values, since 76% (2457/3234) of the commonly predicted chemicals with acidic pKas were concordant (difference ≤ 2 pKa units), while only 46% (1089/2366) of the commonly predicted chemicals with basic pKas were concordant. Similarly, in comparison to DataWarrior data, ACD/Labs seemed to be more specific regarding the acid/basic classification and the ionizables/non-ionizables, while ChemAxon considered most chemicals as ionizables and amphoteric.

**Fig. 8:**
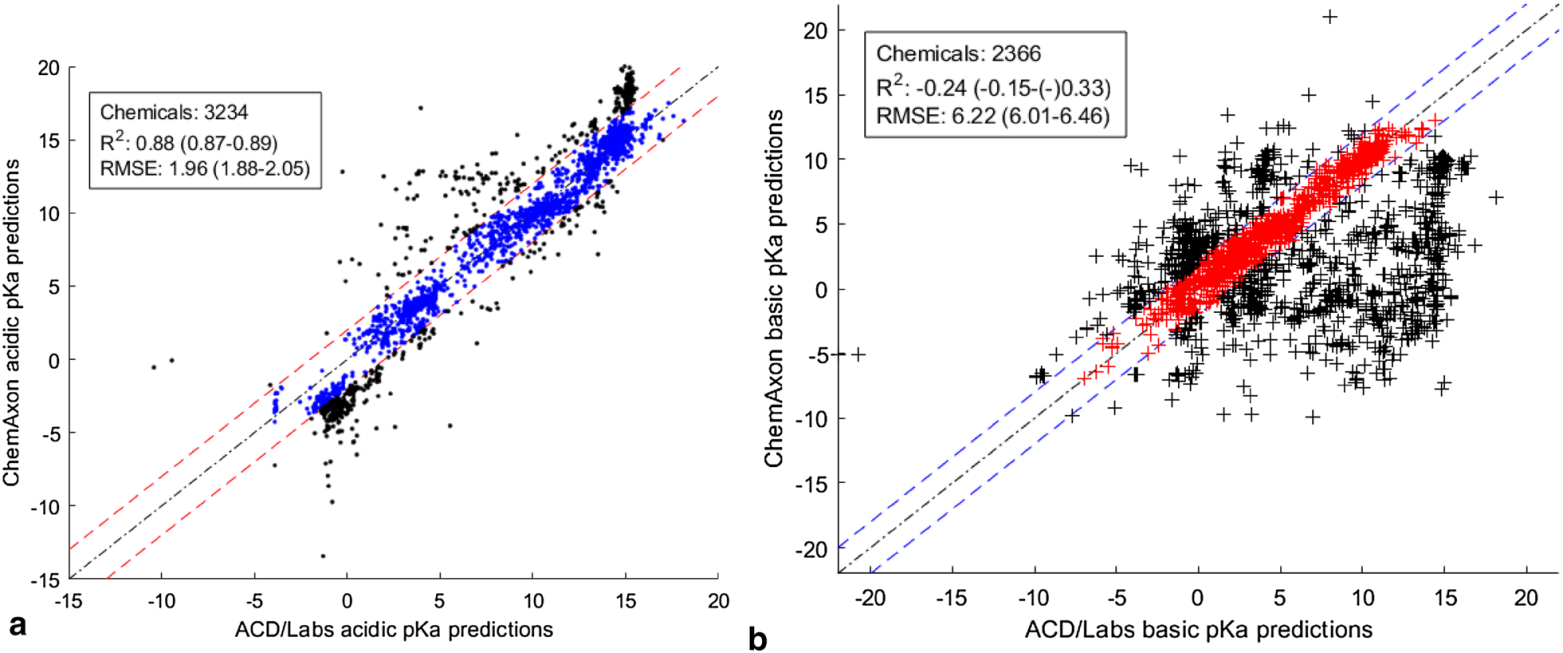
Scatter plots of the ChemAxon and ACD/Labs predictions for the TSCA_active data set. **a** Chemicals predicted to have acidic pKa within 2 pKa units are shown in blue, and chemicals with predicted pKa values differing by more than 2 pKa units are in black. **b** Chemicals with ACD/Labs and ChemAxon predicted basic pKa values falling within 2 pKa units of each other are shown in red, and chemicals with predicted pKa values differing by more than 2 pKa units are in black. Values between parenthesis are the 95% confidence intervals based on a 5000-bootstrapping procedure

Figure 8 shows scatter plots of predictions of acidic and basic pKa values for the TSCA chemicals. The predictions that are within ± 2 pKa units of each other are highlighted in blue for the acidic pKas and red for the basic pKas. The concordance between ACD/Labs and ChemAxon was greatest for the acidic pKa predictions compared to the basic pKa predictions, which showed more divergence. Thus, as discussed above, it was considered to be better to use only the predictions within ± 2 pKa units of each other for the subsequent benchmark analysis. There were 2457 chemicals with pKa predictions within ± 2 pKa units for the chemicals predicted to have acidic pKa and 1089 chemicals with pKa predictions within ± 2 pKa units for chemicals predicted to have a basic pKa (Fig. 8). The ACD/Labs and ChemAxon pKa predictions were averaged for these chemicals and used as benchmark datasets.

The SVM, XGB, and DNN models developed in this work were used to predict pKa values of the TSCA chemical data set of 8904 chemicals for further benchmarking. The SVM model was implemented in OPERA with a kNN classifier to determine if a chemical would have an acidic, basic, or amphoteric pKa(s). In addition, OPERA provided an AD and accuracy assessment. Neither the XGB or DNN models predicted if a chemical would have an acidic or basic pKa, as shown in Table 11, so all chemicals were predicted using both the acidic and basic models.

**Table 11:** Predictions of pKa for the 8904 TSCA chemicals

	Acidic pKa predictions	Basic pKa predictions	Non-ionizable
OPERA (SVM + kNN)	3141	2201	3696
Chemicals within OPERA AD	2374 (76%)	1850 (84%)	3201 (87%)
XGB	8904	8904	Not applicable
DNN	8904	8904	Not applicable

**Fig. 9:**
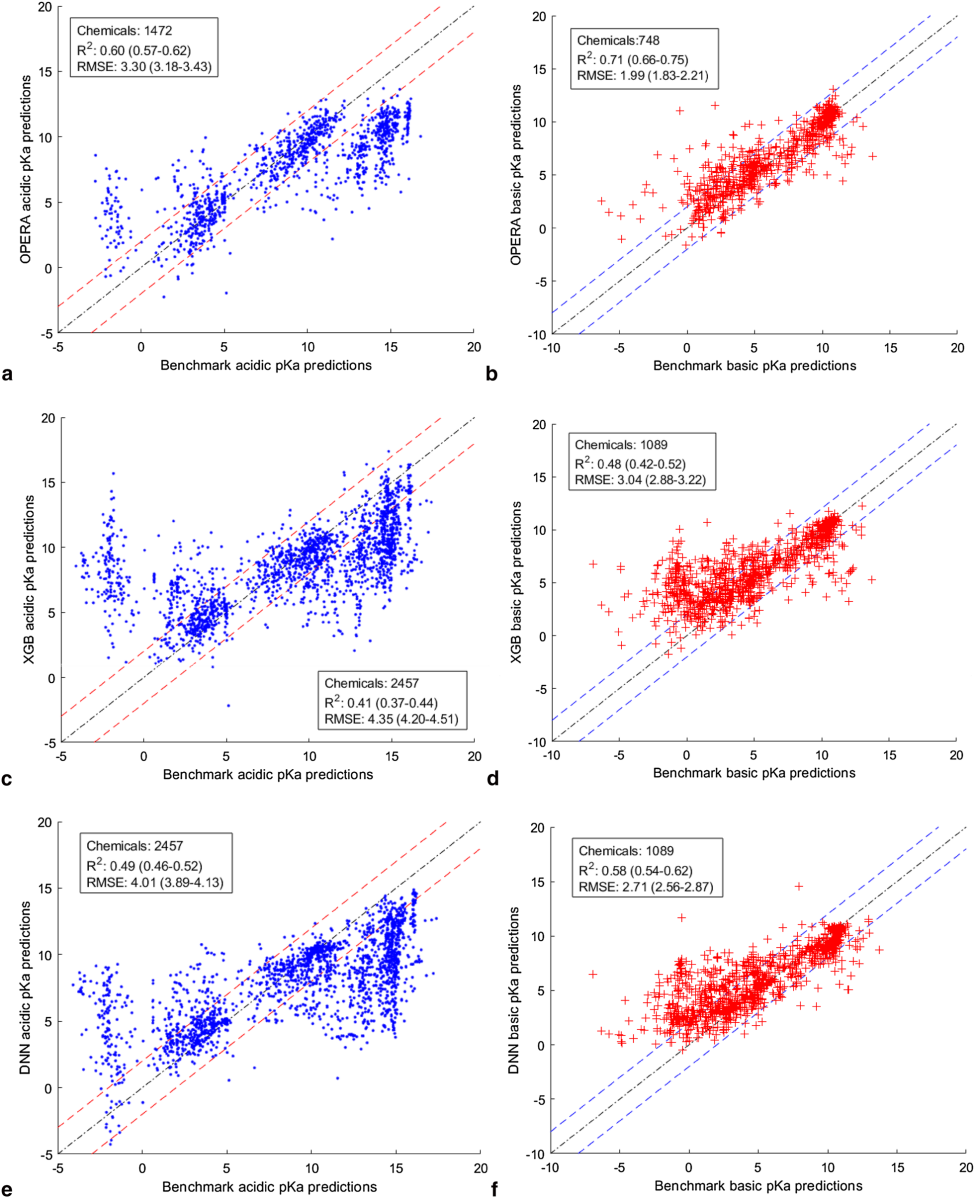
Scatter plots showing the acidic and basic pKa predictions by OPERA, XGB, and DNN, compared to the averaged ChemAxon and ACD/Labs predictions for the benchmark list of chemicals. **a** Benchmark acidic pKa predictions vs OPERA acidic pKa predictions. **b** Benchmark basic pKa predictions vs OPERA basic pKa predictions. **c** Benchmark acidic pKa predictions vs XGB acidic pKa predictions. **d** Benchmark basic pKa predictions vs XGB basic pKa predictions. **e** Benchmark acidic pKa predictions vs DNN acidic pKa predictions. **f** Benchmark basic pKa predictions vs DNN basic pKa predictions. Values between parenthesis are the 95% confidence intervals based on a 5000-bootstrapping procedure

Comparing the data in with the ACD/Labs predictions in Table 10 shows that the OPERA predictions were highly concordant with those of ACD/Labs in terms of the number of acidic and basic classifications. Most of these predictions were within the AD of the OPERA models. The predictions of the OPERA, XGB, and DNN models using the benchmark acidic and basic datasets are plotted in Fig. 9 along with the R^2^ and RMSE to assess the concordance in pKa values. For OPERA, only the overlapping predictions within the AD are plotted.

Figure 9 shows a reasonable concordance between the three models and the two benchmark datasets. However, the concordance with the basic benchmark data set was higher than the acidic dataset.

When the whole TSCA_active list was considered (Fig. 8), the discordance between ACD/Labs and ChemAxon was higher for the basic dataset compared to the acidic dataset. However, for the benchmark datasets, which includes only predictions within 2 pKa units of each other, the opposite was noticed, namely that the basic dataset showed better concordance with the OPERA, XGB and DNN models.

OPERA showed better concordance than the XGB and DNN models with the benchmark acidic and basic pKa predictions as evidenced by the R^2^ and RMSEs in Fig. 9. This can be explained by the fact that the models in this work were trained on DataWarrior datasets that were not fully concordant with ACD/Labs and ChemAxon in terms of acidic/basic and ionizables/non-ionizables. In addition, the DataWarrior chemicals define the chemical space of the OPERA models, thus not all TSCA_active chemicals (Table 11) and the benchmark subset are all within OPERA’s AD.

As shown in Tables 7 and 8, the number of overlapping predictions between the two tools was higher than the number of pKa values in DataWarrior, although not all DataWarrior acidic and basic datasets were predicted as such by the two tools. The same trend is noticed with the TSCA_active chemicals as shown in Tables 10 and 11.

Table 12 summarizes the effect of defining the AD using the statistics of OPERA predictions for the benchmark dataset. As expected, concordance for the predictions outside of the AD was much lower than that for predictions inside the AD. For the acidic dataset, the difference between the RMSE values for chemicals inside and outside the AD was 2.11, which is substantial. Thus, as would be expected, excluding the predictions outside of the AD improved the statistics of the models since the predictions within the AD can be considered more accurate than those outside the AD.

**Table 12:** Effect of AD definition on OPERA’s concordance metrics

	Acidic benchmark dataset (2457)			Basic benchmark dataset (1089)		
	All overlapping	Inside AD	Outside AD	All overlapping	Inside AD	Outside AD
Number of chemicals	1847	1472	375	853	748	105
R^2^	0.53	0.60	0.18	0.68	0.71	0.56
RMSE	3.82	3.3	5.41	2.17	1.99	3.18

The other reason for the lower concordance between the models developed in this work and the benchmark dataset is due to the high number of discordant predictions at both extremes of the benchmark acidic pKa predictions (Fig. 9a, c, e). This discordance is not only due to the difference between the three models and the benchmark data, but also between ACD/Labs and ChemAxon predictors. As seen in Fig. 8a, the predictions of the two commercial tools begin to diverge at the extremities of the scatter plots for the acidic dataset. The pKa range where these two tools are the most concordant is [0–14], which is also the range for most of the DataWarrior acidic pKa values (Fig. 3). Thus, the benchmark acidic dataset can be reduced to the range of DataWarrior acidic pKa values [0–14] that was used to train the three models developed in this work. By excluding the extreme acidic pKa values, the benchmark dataset was reduced from 2457 to 1629 chemicals.

Likewise, the extreme values were removed from the basic benchmark dataset by restricting the pKa values to [− 2, 12] which is the range of the DataWarrior basic pKas. The resulting basic benchmark dataset was reduced from 1089 to 1047 chemicals.

The concordance statistics between the three models and the reduced benchmark datasets are summarized in Table 13.

**Table 13:** Comparison of models developed in this work with commercial programs in predicting benchmark data pKas

	Reduced acidic benchmark dataset (1629)			Reduced basic benchmark dataset (1047)		
	OPERA	XGB	DNN	OPERA	XGB	DNN
Overlapping chemicals	1059	1629	1629	731	1047	1047
R^2^	0.57	0.48	0.48	0.73	0.50	0.60
RMSE	2.42	2.88	2.90	1.81	2.79	2.51

As expected, by excluding the extreme values that are the source of divergence between the commercial tools and are absent in DataWarrior, the overall concordance between the benchmark datasets and the three models increased. This increase is clear for the acidic dataset after removing the 828 extreme pKa values, while only 42 pKa values were removed from the basic dataset. The concordance improvement was higher for the XGB and DNN models in comparison to OPERA. For OPERA, ~ 50% of the extreme values were already excluded by the AD or predicted to be non-ionizable. This explains why the chemicals outside of the AD had lower concordance with the benchmark dataset. Removing the extreme values from the acidic benchmark dataset also decreased the difference in RMSE between the three models with the benchmark dataset. The DNN, XGB, and OPERA models showed about the same performance statistics (R^2^ and RMSE) with the reduced acidic benchmark dataset.

This benchmark analysis and comparison revealed many differences among all models with respect to the predictions of the pKa values and how chemicals are predicted to have an acidic or basic pKa. Differences were noted among the models developed in this work as well as between the commercial tools, and this applied to both analyses based on the DataWarrior and the benchmark dataset. The DNN and XGB models do not predict whether a chemical will have an acidic or basic pKa, unlike ACD/Labs ChemAxon and OPERA. Thus, while OPERA can be applied directly to large numbers of chemicals to identify the ionizables then predict the relative acidic and basic pKas in batch mode, the DNN and XGB models provide the users with the flexibility to manually select ionizable chemicals, applying expert judgment if dealing with a limited number of chemicals, or to plug in external ionization algorithms. Since the three resulting models from this work are QSAR models trained on a dataset with only the strongest acidic and basic pKas, they do not provide pKas for all ionization sites for multiprotic compounds.

For OPERA (release v2.0), the pKa model currently available on the Github repository is available as both a command line module and in the form of a user-friendly graphical interface [45]. The pKa predictions in OPERA can also be used to make logD estimates for physiological pH values of interest, specifically pH 5.5 and pH 7.4. All OPERA predictions are provided with AD and accuracy estimates as well as experimental and predicted values for the nearest neighboring chemicals as shown on the EPA Dashboard prediction reports and explained in Mansouri et al. [27].

## Conclusions

Open source pKa prediction models using SVM, XGB, and DNN algorithms were built using the freely available DataWarrior pKa data set. The chemical structures contained in this list were curated and standardized for modeling, then associated with chemical identities from the EPA’s DSSTox database. Prediction models were trained on a subset containing 75% of the full data set and tested on the remaining 25%. Acidic and basic pKa values were modeled separately. Performance of the models for predicting the test set pKas was reasonably good, with RMSE values as low as 1.50 and R^2^ values up to 0.80.

Predictions from commercial software produced by ACD/Labs and ChemAxon were compared to experimental values from DataWarrior acidic and basic datasets. The concordance of the two tools with the DataWarrior values was similar. However, we discovered that the most concordant predictions between ACD/Labs and ChemAxon were also the most concordant with the experimental data from DataWarrior. This can be considered as an indication of the accuracy of the predictions of the two commercial tools for the DataWarrior datasets, namely that their predictions are more accurate when both predictions are within ± 2 pKa units and more inaccurate as they diverge (> 2 pKa units difference). Based on this observation, the concordant predictions of the two commercial tools (within 2 pKa units) were averaged and used as a benchmark dataset for the three open-source models developed in this work.

The benchmark analysis of the three models was conducted on a subset of the TSCA_active chemicals downloaded from the EPA CompTox Chemicals Dashboard. These chemicals had no experimental pKa values. However, based on our observations, the benchmark datasets resulting from the concordant ADC/Labs and ChemAxon predictions were considered close enough to experimental values based on the comparison to DataWarrior datasets. Our results indicate that the extreme acidic and basic predictions outside the range of [0–14] and [− 2 to 12] for the acidic and basic datasets respectively for the two commercial tools might be associated with lower accuracy. This limitation is also applicable to the three models developed in this work, since they were trained on DataWarrior data that is mostly in [0–14] and [− 2 to 12] ranges for the acidic and basic pKas, respectively.

The two comparison studies conducted in this work, based on the experimental data provided by DataWarrior as well as the benchmark set from the TSCA chemicals, revealed a number of differences among all models. The differences are related to the accuracy of the pKa values predicted, as well as the classification of chemicals into acidic, basic, or amphoteric forms. Although there was a certain level of concordance among the different predictions, it is clear is that pKa is a challenging property to model. While many methods for predicting pKa have been developed for restricted chemical spaces, we believe that we have developed fully open data and open-source methods for predicting the most acidic and basic pKas for a wide range of chemicals and pKa values. An additional output from this work is an improved version of the DataWarrior pKa data set obtained by standardizing the chemical structures and registering them into the DSSTox database. Finally, all model predictions have been available via the EPA CompTox Chemicals Dashboard for further use by the scientific community.

## Future work

This research produced a pKa data set curated using EPA’s standard approaches to producing training sets for the pKa predictions to be delivered via the EPA CompTox Chemicals Dashboard. Scientists within the EPA are taking advantage of pre-computed values from different models, accessible via the Dashboard, to source predicted data for large numbers of chemicals by using the batch search [67]. With an OPERA model for pKa and logD prediction now available, the entire collection of QSAR-ready standardized structures derived from the 765,000 chemical substances associated with the Dashboard was run through OPERA. The resulting pKa and logD values will be published on the associated chemical properties pages, and will include a detailed calculation report showing the applicability domain details and nearest neighbors used for prediction. As with all other OPERA models, a QSAR Model Report Format (QMRF) detailing the OPERA pKa prediction model will be available from the Dashboard (for example, logP: https://comptox.epa.gov/dashboard/dsstoxdb/download_qmrf_pdf?model=22&model_name=OPERA_LogP).

The DNN and XGB models do not predict whether a chemical will have an acidic or basic pKa, unlike ACD/Labs, ChemAxon, and OPERA. In future work, both XGB and DNN will be modified to predict whether a chemical will have an acidic or basic pKa.

Predicted pKa values will be available for modeling efforts such as high-throughput toxicokinetics [68] for potential application to toxicity prediction [69], and even to support chemical identification using predicted retention times to aid with candidate ranking in non-targeted screening by mass spectrometry. The importance of logD for the prediction of retention time has already been noted a number of times including in our own studies [35].

The EPA CompTox Chemicals Dashboard presently delivers real time prediction capabilities whereby a user can draw a chemical structure in a web-based drawing editor and predict a number of physicochemical and toxicological endpoints [70]. The integration of OPERA models to allow for real-time prediction is presently underway, and the inclusion of the OPERA pKa and logD predictive models is already planned. Since all OPERA models are free and open-source, as are the other models discussed in the publication, the community will have access to multiple pKa models that they can integrate into their own software applications.

## Supplementary information

**Additional file 1.** Original and curated pKa data used for modeling.

**Additional file 2.** Additional data analysis and modeling information.

**Additional file 3.** DataWarrior pKa data registered in DSSTox database.

**Additional file 4.** Predictions of ACD/Labs and ChemAxon for DataWarrior chemcials.

**Additional file 5.** Predictions of ACD/Labs, ChemAxon and the three models on the benchmark data.

## Data Availability

All data sets and code are available as Additional files attached to this paper and on GitHub: https://github.com/NIEHS/OPERA

## Abbreviations

AD: applicability domain
BA: balanced accuracy
DNN: deep neural network
EPA: U.S. Environmental Protection Agency
GA: genetic algorithms
kNN: k-nearest neighbor
Ka: acid dissociation constant (also called protonation or ionization constant)
logD: pH-dependent lipid-aqueous partition coefficient
logP: lipid-aqueous partition coefficient for non-ionizable substances (also expressed as logK_ow_)
OPERA: Open Structure–Activity/Property Relationship App
PK: pharmacokinetic
pKa: − log10 Ka
QSAR: quantitative structure–activity relationship
QSPR: quantitative structure–property relationship
R^2^: coefficient of determination
Q^2^: coefficient of determination in cross-validation
r^2^: coefficient of correlation
RMSE: root-mean-squared error
SVM: support vector machines
TSCA: Toxic Substances Control Act
XGB: extreme gradient boosting
