# Supplementary material for: Open-source QSAR models for pKa prediction using multiple machine learning approaches
Source: J Cheminform. 2019 Sep 18;11:60. doi: 10.1186/s13321-019-0384-1 (PMC6749653; doi:10.1186/s13321-019-0384-1)
Supplement: Supplementary file 2 — Additional file 2. Additional data analysis and modeling information. [file 13321_2019_384_MOESM2_ESM.docx]

**Additional Information For "Open Source QSAR Models For pKa Prediction Using Multiple Machine Learning Approaches"**

**DATE: 6 August 2018**

#

# pKa data

#
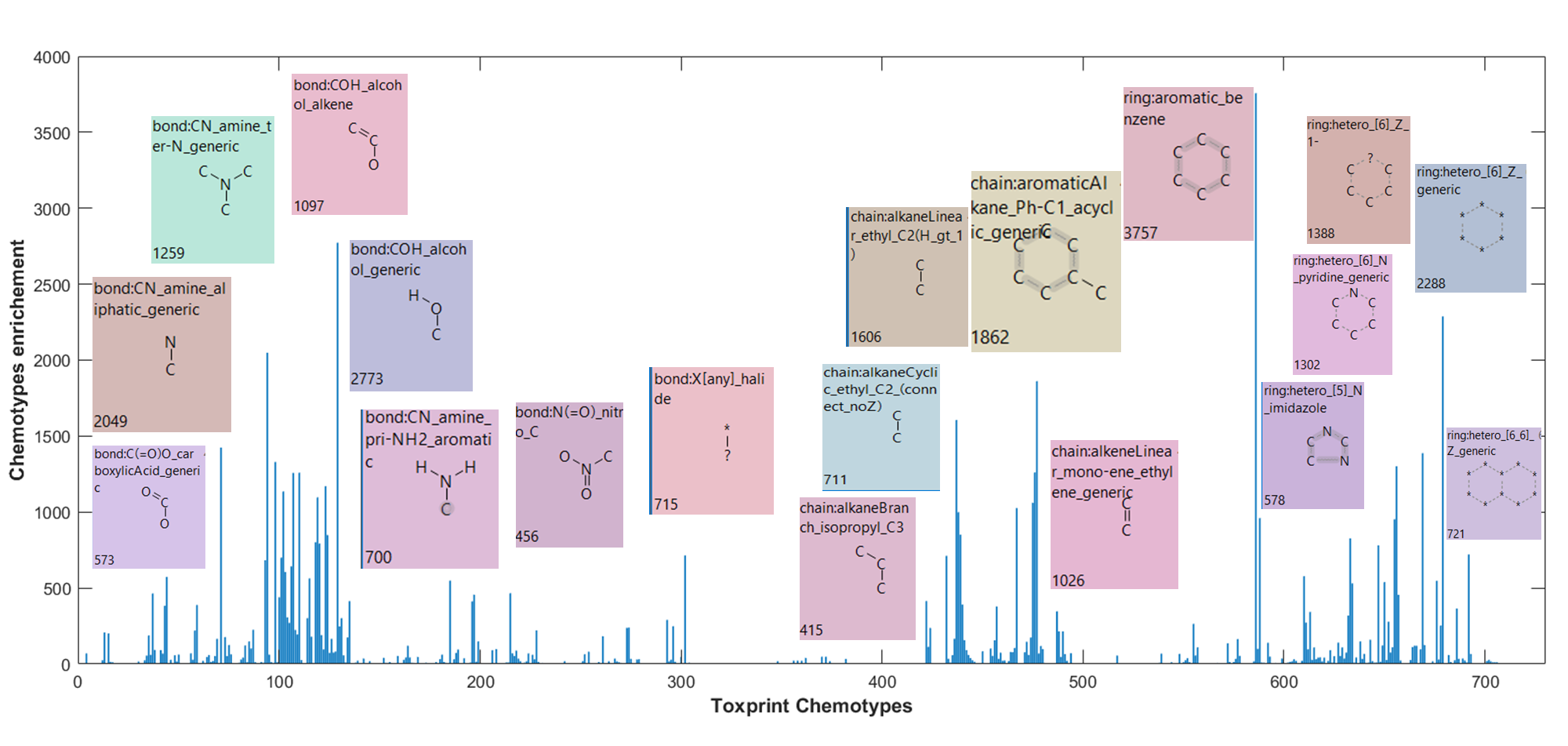


# Methods

## Support Vector Machines (SVM)

### Additional SVM Details

SVM algorithms search for the support vectors that give the best separating hyperplane using a kernel function. During optimization, SVM search the decision boundary with maximal margin among all possible hyperplanes, where the margin can be intended as the distance between the hyperplane and the closest point for both classes. This procedure was carried out by means of a kernel based on a radial basis function.

### R^2^ And Q^2^ Calculations

These two coefficients were calculated in equations 1 and 2 as follows:


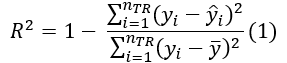


where
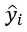
 and y_i_ are the estimated and observed responses of the *i_th_* element, respectively.
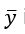
 is the mean and n and  is the number of training compounds.


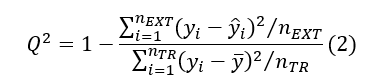


where n_EXT_ is number of test compounds and n_TR_ is the number of training compounds,
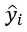
 and y_i_ are the estimated and observed responses, respectively, and
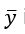
 is the mean.

### Genetic Algorithms (GAs)

GAs start from an initial random population of chromosomes, which are binary vectors representing the presence or absence of molecular descriptors. Then an evolutionary process is simulated to optimize a defined fitness function and new chromosomes are obtained by coupling the chromosomes of the initial population with genetic operations (crossover and mutation). The fitness function used was the Q2 calculated in 5-fold cross-validation.

## Extreme Gradient Boosting

### Data Preparation, 5-Fold Cross Validation and Variable Importance

The binary fingerprints were converted to the R logical data type and were not run as integer (0 or 1) as small variations were seen between the logical and integer metrics.

The caret function trainControl was used to specify training parameters for the caret train function. Five-fold cross-validation repeated five times was set using the trainControl function to assess the training data model performance. RMSE (Root Squared Mean Error) was the metric optimized by the caret train function. RMSE and R-Squared were used to assess model performance. Both Pearson Correlation Coeffient (r^2^) and the determination of

pKa outliers were identified and removed using the Interquartile Range (IQR)^^[[1]](#footnote-1)^^. Outliers were defined as those pKa values below Q1 - 1.5 * IQR and values above Q3 + 1.5 * IQR. This resulted in 0.29 - 0.90% of chemicals with a binary fingerprint being removed and between 0.70 - 1.9% of chemicals using 1D/2D descriptors.

### Variable Importance

A Variable Importance and a r^2^ plot was generated for each XGB model. Variable importance shows the specific features which are contributing most strongly to model performance. The Variable Importance and r^2^ plots for the best performing model using the testing dataset are given in Figure S1 and Figure S2.


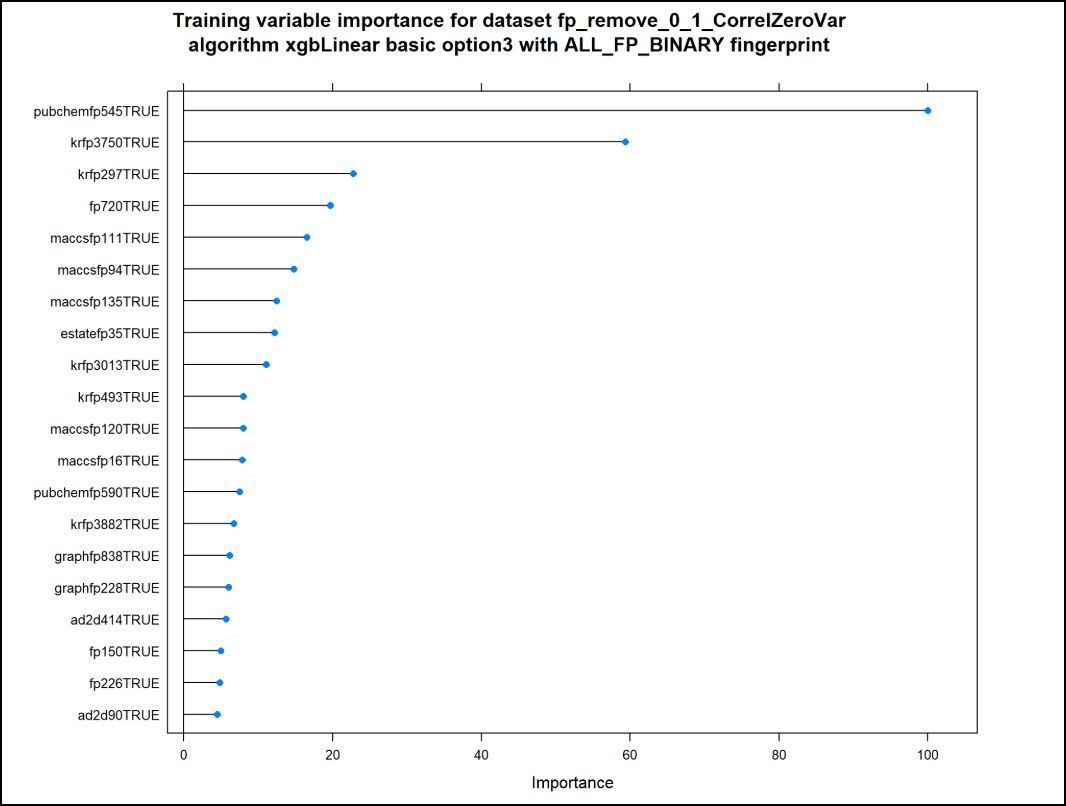


Figure S1. Variable Importance for the model which gave the best overall performance (testing dataset, chemicals with a basic pKa, the 8 concatenated fingerprints with Option 3 data).


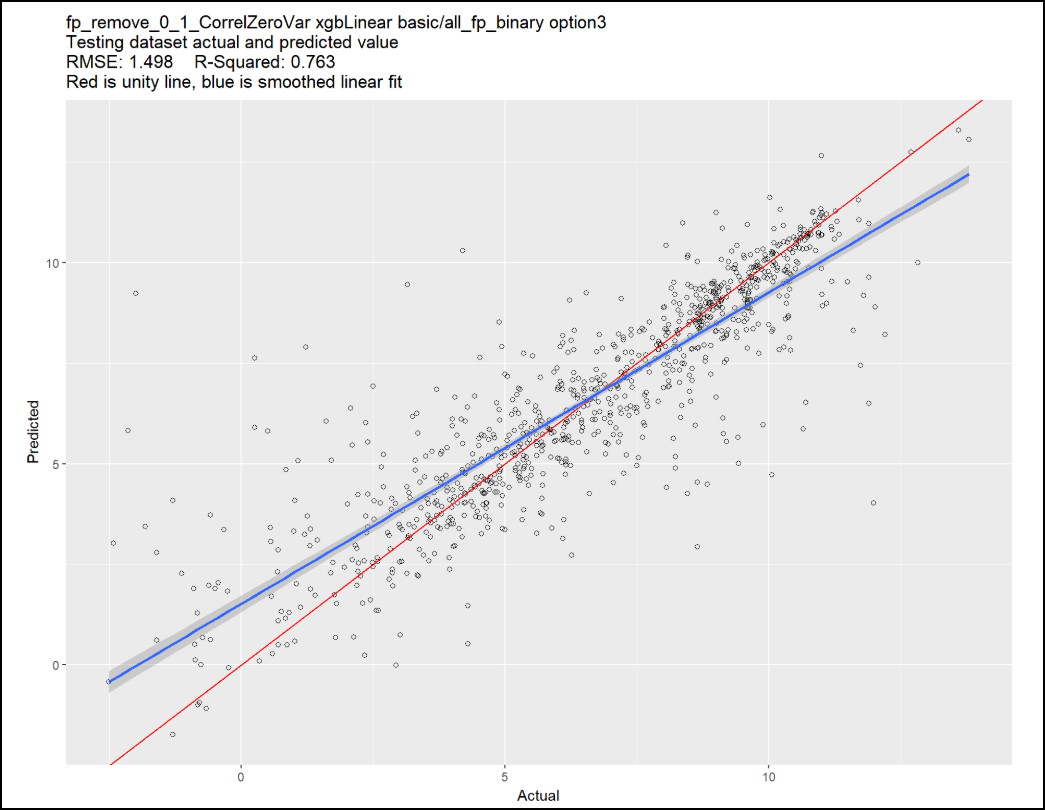


Figure S2. Actual and predicted values for the best model (testing dataset, chemicals with a basic pKa, the 8 concatenated fingerprints with Option 3 data). The unity line is shown in red and a smoothed linear fit is given in blue. RMSE and r^2^ (Pearson) values are given in the plot.

and Figure S3 shows the residuals for the XGB model for chemicals with an acidic pKa.


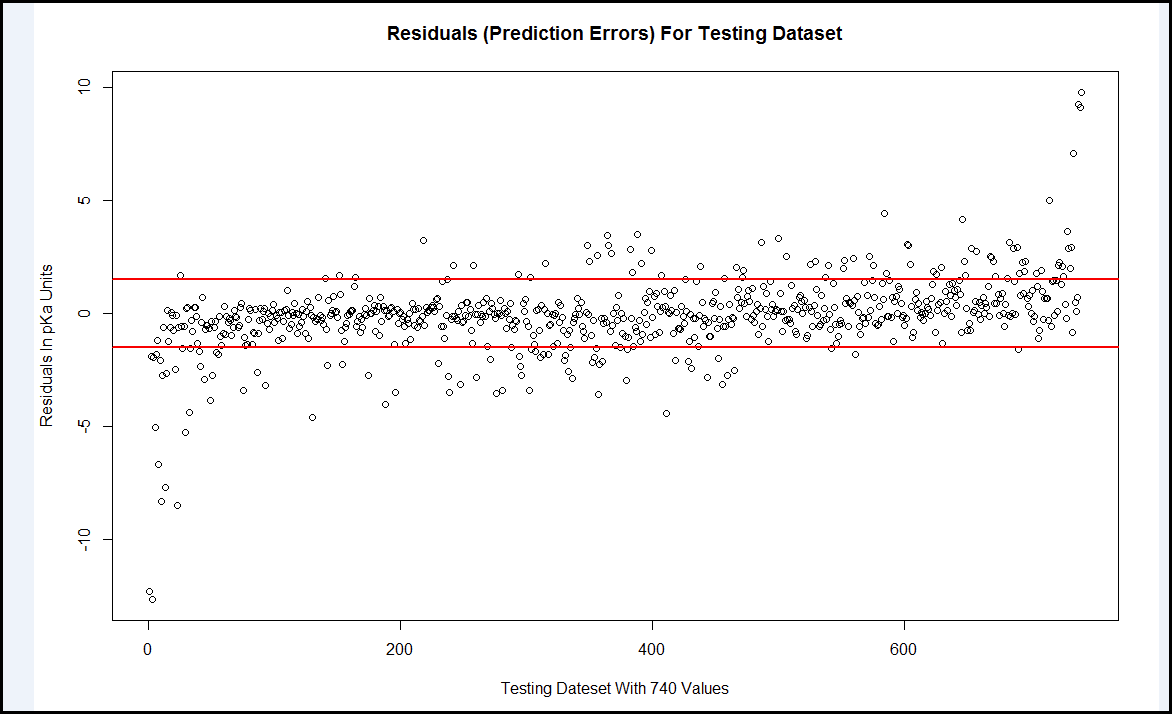


Figure S3. Residuals (prediction errors) for the test set with the best RMSE using Extreme Gradient Boosing. The RMSE value for this model was 1.68. Red lines indicate ± 1 RMSE, and this interval included 81% of the RMSE values.

### The Best Model found using Extreme Gradient Boosting was Improved by using a Simple Deep Neural Network

The best performance using XGB occurred for chemicals with a basic pKa using the Option 3 dataset after removing (i) features that were all 0's or all 1's, (ii) features that were highly-correlated and (iii) features that showed little variance.

Tensorflow and keras were used to construct a feed-forward Deep Neural Network (DNN) using this data. The model details are as follows: (1) 256 nodes, (2) 64 nodes, (3) batch normalization, (4) dropout, (5) 32 nodes, (6) batch normalization, (7) dropout and (8) 1 node.

The dataset was examined with the Adam, Nadam, Rmsprop and SGD optimizers. The Adam optimizer gave the best RMSE results as shown in Table 1.

| **Optimizer** | **Train RMSE** | **Train r-Squared (Pearson Correlation)** | **Test RMSE** | **Test r-Squared (Pearson Correlation)** |
| --- | --- | --- | --- | --- |
| adam | 0.352 | 0.987 | 1.382 | 0.814 |
| nadam | 0.312 | 0.990 | 1.403 | 0.808 |
| rmsprop | 0.332 | 0.989 | 1.442 | 0.798 |
| sgd | 0.416 | 0.982 | 1.467 | 0.791 |

Table 1. Neural Network model performance with different optimizers.

The Deep Learning model performance was superior to the best model using Extreme Gradient Boosting, improving RMSE by 8%. A Jupyter notebook on the GitHub site contains the Python code.

1. <https://en.wikipedia.org/wiki/Interquartile_range> [↑](#footnote-ref-1)
